# Response to Letter to the Editors regarding ‘Efficacy of psychosocial interventions to reduce alcohol use in comorbid alcohol use disorder and alcohol-related liver disease: a systematic review of randomized controlled trials’

**DOI:** 10.1093/alcalc/agae004

**Published:** 2024-01-27

**Authors:** Sofia Hemrage, Eileen Brobbin, Paolo Deluca, Colin Drummond

**Affiliations:** Department of Addictions, Institute of Psychiatry, Psychology and Neuroscience, King’s College London, 4 Windsor Walk, London SE5 8BB, United Kingdom; Department of Addictions, Institute of Psychiatry, Psychology and Neuroscience, King’s College London, 4 Windsor Walk, London SE5 8BB, United Kingdom; Department of Addictions, Institute of Psychiatry, Psychology and Neuroscience, King’s College London, 4 Windsor Walk, London SE5 8BB, United Kingdom; Department of Addictions, Institute of Psychiatry, Psychology and Neuroscience, King’s College London, 4 Windsor Walk, London SE5 8BB, United Kingdom

Dear Editors,

We thank Dr Wei for reading, considering, and taking the time to provide feedback on our findings published under ‘Efficacy of psychosocial interventions to reduce alcohol use in comorbid alcohol use disorder and alcohol-related liver disease: a systematic review of randomized controlled trials’ (Hemrage et al. 2023). We also wish to thank you for the opportunity to further clarify the points raised in Dr Wei’s letter. We appreciate the occasion to engage in constructive discussions that further the scientific method.

Dr Wei requested further context to the risk of bias assessment for the included randomized controlled trials. We conducted an assessment of the risk of bias using the Cochrane’s Risk-of-Bias 2 tool, as the gold standard to determine the risk of bias in randomized controlled trials (Sterne et al. 2019). The tool includes criteria such as random sequence generation and allocation concealment (selection bias) and incomplete outcome data (attrition bias). Similar to Kelly and colleagues (Kelly et al. 2020), blinding of participants and personnel delivering the intervention is not always possible given the nature of psychosocial interventions. Despite being of moderate certainty, the findings of our systematic review grant further evidence to the scope of psychosocial interventions to reduce harm in alcohol-related liver disease. Given the methodological limitations of some of the published research on this topic, this points to the need for more high-quality studies on psychosocial interventions in alcohol-related liver disease in the future to enrich the evidence base.

We agree with Dr Wei, that given the physical and mental comorbidities inherent to alcohol-use disorder and alcohol-related liver disease, treatment approaches should consider and tailor interventions to the unique therapeutic needs of this clinical population. Despite the effectiveness of pharmacotherapy for the management of alcohol-use disorder, patients with comorbid alcohol-use disorder and alcohol-related liver disease often present different responses to medication given the heterogeneity in their hepatic profiles and respective pharmacodynamic processes. There are also several contraindications for various pharmacotherapies in alcohol-related liver disease due to abnormalities in liver function, especially for disulfiram and naltrexone (Thursz and Lingford-Hughes 2023). Hence, there is an important role in the use of psychosocial interventions to improve patient outcomes and reduce alcohol-related harm in comorbid alcohol-use disorder and alcohol-related liver disease, which was the focus of our systematic review. Where clinically possible, these psychosocial interventions should be used in conjunction with effective pharmacotherapies to maximize clinical effectiveness, although there is currently a lack of research on combined therapies specifically in alcohol-related liver disease (Thursz and Lingford-Hughes 2023).

Motivational enhancement therapy was consistently found to be effective in the included randomized controlled trials in addressing comorbid alcohol-use disorder and alcohol-related liver disease by improving both reduction and abstinence outcomes. While a study trialling cognitive behavioural therapy saw a significant increase in abstinence, their implications should be noted with caution given age baseline differences and protocol deviations (Willenbring and Olson 1999).

Ultimately, we concur with Dr Wei’s call for future research to build upon evidence-based interventions that address this unmet need, to improve patient outcomes among this clinical population, particularly the need for care which effectively integrates psychosocial, pharmacological and hepatology interventions.
